# HNRNPC regulates RhoA to induce DNA damage repair and cancer‐associated fibroblast activation causing radiation resistance in pancreatic cancer

**DOI:** 10.1111/jcmm.17254

**Published:** 2022-03-11

**Authors:** Ning Xia, Nannan Yang, Qungang Shan, Ziyin Wang, Xiaoyu Liu, Yingjie Chen, Jian Lu, Wei Huang, Zhongmin Wang

**Affiliations:** ^1^ Department of Radiology Ruijin Hospital Luwan Branch Shanghai Jiao Tong University School of Medicine Shanghai China; ^2^ Department of Interventional Radiology Ruijin Hospital Shanghai Jiao Tong University School of Medicine Shanghai China

**Keywords:** DNA damage repair, HNRNPC, pancreatic cancer, radiation resistance, RhoA

## Abstract

Pancreatic cancer (PC) is one of the most lethal types of cancer due to its asymptomatic nature in the early stages and consequent late diagnosis. Its mortality rate remains high despite advances in treatment strategies, which include a combination of surgical resection and adjuvant therapy. Although these approaches may have a positive effect on prognosis, the development of chemo‐ and radioresistance still poses a significant challenge for successful PC treatment. Heterogeneous nuclear ribonucleoprotein C1/C2 (HNRNPC) and RhoA have been implicated in the regulation of tumour cell proliferation and chemo‐ and radioresistance. Our study aims to investigate the mechanism for HNRNPC regulation of PC radiation resistance via the RhoA pathway. We found that HNRNPC and RhoA mRNA and protein expression levels were significantly higher in PC tissues compared to adjacent non‐tumour tissue. Furthermore, high HNRNPC expression was associated with poor patient prognosis. Using HNRNPC overexpression and siRNA interference, we demonstrated that HNRNPC overexpression promoted radiation resistance in PC cells, while HNRNPC knockdown increased radiosensitivity. However, silencing of RhoA expression was shown to attenuate radiation resistance caused by HNRNPC overexpression. Next, we identified RhoA as a downstream target of HNRNPC and showed that inhibition of the RhoA/ROCK2‐YAP/TAZ pathway led to a reduction in DNA damage repair and radiation resistance. Finally, using both in vitro assays and an in vivo subcutaneous tumour xenograft model, we demonstrated that RhoA inhibition can hinder the activity of cancer‐related fibroblasts and weaken PC radiation resistance. Our study describes a role for HNRNPC and the RhoA/ROCK2‐YAP/TAZ signalling pathways in mediating radiation resistance and provides a potential therapeutic target for improving the treatment of PC.

## 1 INTRODUCTION

Pancreatic cancer (PC) is the eleventh most common cancer worldwide and seventh leading cause of cancer mortality in both men and women due to its poor prognosis and late‐stage diagnosis.^1^ Early‐stage PC is typically asymptomatic, while late‐stage symptoms, such as abdominal pain, jaundice and pruritus, are non‐specific,^2^ making correct diagnosis especially challenging. Current diagnostic methods rely on a combination of computed tomography scans, magnetic resonance imaging, ultrasound imaging, cholangiopancreatography, positron emission tomography, angiography, blood tests and biopsy results.^3^ PC risk factors include smoking, alcohol, obesity, certain dietary habits, occupational exposure to metal and pesticides, advanced age, male gender, ethnicity, diabetes mellitus, family history, genetic factors, ABO blood group, pancreatitis and some chronic infections.^4^

While some advances in treatment strategies have been made, the five‐year survival rate for PC remains low at 9%.^5^ Although surgical resection and adjuvant therapy are common treatment strategies for PC depending on the stage of disease,^6^ recent studies have demonstrated the benefits of applying a neoadjuvant approach, which include controlling potential micrometastases (usually present at the time of diagnosis), and determining which patients would benefit from surgery while sparing unsuitable patients from major surgical intervention.^7^, ^8^ Current first‐line chemotherapeutic regimens for metastatic pancreatic cancer patients involve treatment with either FOLFIRINOX (folinic acid, 5‐fluorouracil, irinotecan and oxaliplatin) or gemcitabine plus nab‐paclitaxel,^9^ with recent studies indicating that FOLFIRINOX may be the better option.^10^ However, the eventual and inevitable development of chemo‐ and radioresistance significantly limits their treatment efficacy.^9^, ^11^ PC cells are particularly prone to developing endogenous and exogenous resistance to gemcitabine.^5^ Therefore, it is important to investigate additional agents that might enhance chemotherapy efficacy in drug‐sensitive cells and reduce resistance in drug‐resistant cancer cells.

Previous studies have reported elevated levels of heterogeneous nuclear ribonucleoproteins C1/C2 (HNRNPC) in some cancer cells, while HNRNPC knockdown resulted in significant arrest of cell proliferation and tumour growth in breast cancer.^12^ Conversely, elevated levels of HNRNPC may contribute to a metabolic environment that is beneficial for proliferation.^13^ A more in‐depth investigation has established an association between mRNA‐binding protein hnRNP‐Q1 and small GTPase RhoA. In particular, hnRNP‐Q1 is involved at the cellular level in RhoA‐dependent cellular morphogenesis and the molecular level as a RhoA mRNA translation repressor.^14^ These findings also suggested that the 3′ untranslated region (UTR) of RhoA mRNA is essential for hnRNP‐Q1‐mediated regulation of RhoA expression. Furthermore, previous studies have also shown that RhoA silencing by small‐interfering RNA (siRNA) reduces proliferation and migration of tumour cells and may improve the cytotoxic effect of chemotherapy in human colon cancer cells, thereby reversing chemoresistance.^15^ Modulation of RhoA activity has been shown to sensitize cells to γ‐radiation by attenuating the DNA damage response and repair pathways.^16^

Rho‐associated protein kinases such as ROCK2 are considered to be key Rho downstream effectors, while ROCK inhibitors have anti‐tumour properties.^17^ In fact, RhoA/ROCK signalling may be important for radiation resistance, with ROCK2 in particular having a role in the regulation of cell division.^18^ ROCK2 has also been associated with chemotherapy resistance, with inhibition of ROCK2 signalling sensitizing gemcitabine‐resistant PC cells to gemcitabine‐mediated DNA damage.^19^ In addition, YES‐associated protein (YAP), which is a downstream target of RhoA signalling,^20^ is upregulated in PC and participates in tumorigenesis via epithelial‐mesenchymal transition‐related factors.^21^ Recently, the RhoA/ROCK pathway has been directly linked to the regulation of the YAP/TAZ pathway in fibroblasts.^22^, ^23^ RhoA/ROCK signalling is critically required for the activation of YAP in multiple biological processes^24^ including stem cell development^25^ and atherosclerosis.^26^ However, whether there is a link between HNRNPC and the RhoA/ROCK2 and YAP/TAZ signalling pathways in PC remains unknown.

Cancer‐associated fibroblasts (CAFs) or activated fibroblasts secrete cytokines, growth factors and matrix‐degradation proteins that promote cancer cell proliferation and progression and participate in carcinogenesis, angiogenesis and metastasis.^27^ CAFs are identified by the expression of activated fibroblast markers, such as fibroblast activation protein (FAP), α‐smooth muscle actin (α‐SMA) and fibroblast‐specific protein 1.^28^ Recent studies have suggested that the Hippo pathway and its transcriptional effectors YAP and TAZ are required for fibroblast activation. Nuclear YAP localization is a universal feature of CAFs, and YAP is critical for many aspects of the tumour‐promoting CAF function.^29^ However, although the RhoA/ROCK2‐YAP/TAZ axis has been linked to fibrotic activity and fibroblast differentiation,^23^, ^30^ its role in PC remains unknown.

To determine whether the RhoA/ROCK2‐YAP/TAZ signalling pathways have a role in PC, this study aimed to explore the mechanism for HNRNPC regulation of PC radiation resistance. We hypothesized that (1) HNRNPC positively regulates RhoA post‐transcriptionally, enhances DNA damage repair and promotes PC radiation resistance through the ROCK2/γH2AX pathway and that (2) RhoA activates the YAP pathway, enhances the activation of CAFs and further leads to radiation resistance.

To test our hypothesis, we studied the role of HNRNPC, RhoA, ROCK2, YAP and TAZ in PC tumour and paired adjacent normal tissues and the human PC cell lines BxPC‐3 and Panc‐1 using cell viability, qRT‐PCR, Western blotting, DNA damage repair and fragmentation, RNA immunoprecipitation and immunohistochemistry assays. A xenograft tumour model in nude mice was established to verify our *in vitro* findings. In summary, this study uncovered HNRNPC as a potential therapeutic target for improving PC radiation resistance.

## 2 MATERIALS AND METHODS

### 2.1 Ethics statement

Written informed consent was obtained before collecting PC samples from patients. The Ethics and Institutional Review Committee of the Ruijin Hospital of Shanghai Jiao Tong University of China approved the study, which was conducted in accordance with the ethical standards conveyed in the Declaration of Helsinki.

### 2.2 Patient samples

Tumour and paired adjacent normal tissues were obtained from PC patients who received treatment at the Ruijin Hospital of Shanghai Jiao Tong University of China. None of these patients received neoadjuvant therapy. The samples were collected and immediately stored for subsequent analysis by quantitative real‐time PCR (qRT‐PCR), Western blotting and immunohistochemistry.

### 2.3 Survival analysis using the Kaplan–Meier Plotter web tool

The online database Kaplan‐Meier plotter (http://kmplot.com/analysis/index.php?p=service) was used to determine the five‐year survival rate of pancreatic cancer patients. Kaplan‐Meier plotter performed survival analyses based on gene expression levels.

### 2.4 Cell lines and culture

Human PC cell lines (BxPC‐3 and Panc‐1) were purchased from the Chinese Academy of Sciences Cell Bank of Type Culture Collection (Shanghai, China). All cell lines were cultured in Dulbecco’s modified Eagle’s medium (DMEM, HyClone, Logan, USA) supplemented with 10% foetal bovine serum (FBS), 100 U/mL penicillin and 100 μg/mL streptomycin. The cultures were incubated at 37°C in a humidified atmosphere containing 5% CO_2_. Pancreatic cancer cells were pretreated with clofibrate for 24 h and then were exposed to different dosages of ionizing radiation using an X‐ray linear accelerator (Rad Source, Suwanee, GA, USA) at a fixed dose rate of 1.15 Gy/min.^31^

### 2.5 Cell transfection

Cells were transfected with HNRNPC and RhoA siRNAs (TINGKE, 100 mM) or pcDNA3.1 expressing HNRNPC (pcDNA3.1+HNRNPC, TINGKE, 100 mM) using Lipofectamine 3000 (Invitrogen, Carlsbad, CA, USA) following the manufacturer’s protocol. The siRNA sequences were as follows: si‐HNRNPC‐1; sense 5′‐3′: CAACGGGACUAUUAUGAUA, antisense 5′‐3′: UAUCAUAAUAGUCCCGUUG; si‐HNRNPC‐2; sense 5′‐3′: GCGCUUGUCUAAGAUCAAAUU, antisense 5′‐3′: AAUUUGAUCUUAGACAAGCGC; NC sense5′‐3′UUCUCCGAACG UGUCACGU, antisense 5′‐3′ACGUGACA CGUUCGG AGAA; and si‐RhoA 5′‐GCCACUUA AUGUAUGUUAC‐3′ (sense strand). Cell transfection was performed in six‐well plates and 2500 ng of siRNA or HNRNPC overexpression vector (HNRNPC‐OE) were added to each well. RNA and protein samples were collected after 2–4 days.

### 2.6 Colony formation assay

Cells were seeded in six‐well plates (500 cells/well) and incubated for 24 h. After treatment, cells were cultured for an additional two weeks in drug‐free medium. Cells were then fixed with 4% formaldehyde, stained with 0.1% crystal violet and the number of colonies was counted.

### 2.7 Cell viability assay

Cells were seeded in 96‐well plates (4 × 10^3^ cells/well). Forty‐eight hours after treatment (0‐, 2‐, 4‐, 6‐ or 8‐Gy radiation), MTT solution was added and the plates were placed in a CO_2_ incubator for 4 h at 37°C. The optical density was measured on a microplate reader at 490 nm. Each group was tested in triplicate.

### 2.8 qRT‐PCR assay

Total RNA was extracted using Fastgen1000 (Fastgen, Shanghai, China) according to the manufacturer’s instructions and reverse transcribed into cDNA with Prime Script RT (Takara, Dalian, China). RhoA and HNRNPC mRNA expression was quantitatively examined using qRT‐PCR as described previously.^32^ The primer sequences were: RhoA primer: F: CAGAAAAGTGGACCCCAGAA; R: GCAGCTGCTCTCGTAGCCATTTC; and HNRNPC primer: F: GCCAGCAACGTTACCAACAA; R: TGAACAGAGCAGCCCACAAT. β‐actin was used as the normalization control. Relative gene expression was determined using the 2^‐ΔΔCt^ method.

### 2.9 Western blot analysis

Total protein was extracted using RIPA lysis buffer (Beyotime, Guangzhou, China), and the protein concentration was determined using a BCA protein assay kit (Pierce, Rockford, IL, USA) according to the manufacturer’s instructions. Equal amounts of protein were separated by SDS‐PAGE, then transferred to polyvinylidene fluoride membranes (Millipore, Burlington, MA, USA). Membranes were subsequently incubated with primary antibodies against HNRNPC (1: 1000, Santa Cruz Biotechnology, sc‐32308, Dallas, TX, USA), RhoA (1: 3000, Abcam, ab54835, Cambridge, UK), ROCK2 (1: 1000, Abcam, ab71598), p‐γ‐H2AX (1: 1000, S139; Abcam, ab26350), α‐SMA (1: 500, Abcam, ab7817), FAP (1: 500, Santa Cruz Biotechnology, sc‐65398) and YAP (1: 1000, Abcam, ab56701), followed by incubation with secondary antibodies bound to horseradish peroxidase. Immunoreactivity was visualized using an ECL Western Blot system (Millipore, Burlington, MA, USA). β‐actin was used as an internal loading control.

### 2.10 RNA immunoprecipitation assay

The RNA immunoprecipitation assay (RIP) assay was conducted with a Magna RIP RNA‐binding protein immunoprecipitation kit (Millipore, Billerica, MA, USA). Cells were first lysed with RIP buffer (Millipore), then lysates were incubated with Sepharose beads (Bio‐Rad, Hercules, CA, USA) pre‐coated with HNRNPC antibody. Immunoglobulin G antibody was used as the control. Finally, the quantity of RhoA in the immunoprecipitated complexes was determined by qRT‐PCR.

### 2.11 Luciferase reporter assay

Wild‐type and mutant RhoA 3′UTRs were cloned into a luciferase vector (Promega, Madison, WI, USA) and co‐transfected with HNRNPC‐OE vector into PC cells using Lipofectamine 3000 transfection reagent. Cells were harvested for luciferase activity analysis after 48 h.

### 2.12 DNA damage repair evaluation

DNA damage repair was detected using a 5‐ethynyl‐2‐deoxyuridine assay kit (EdU; RiboBio, Guangzhou, China) according to the manufacturer’s instructions. Briefly, after treatment for 48 h, cells were mixed with a 20 µM EdU solution for 2 h. After fixation and permeabilization, cells were stained with EdU solution for 30 min followed by Hoechst 33342 for a further 30 min. Images were obtained using a Zeiss AxioPlan II epifluorescence (FluoArc) microscope and processed with ImageJ software (NIH, USA). DNA damage repair was determined by evaluating the proportion of EdU‐positive stained cells.

### 2.13 DNA fragmentation evaluation

A terminal deoxynucleotidyl transferase dUTP nick‐end labelling (TUNEL) assay was performed with an In Situ Cell Death Fluorescent Kit (Roche Diagnostic‐Mannheim, Germany) according to the manufacturer’s instructions. Cells were fixed in 4% paraformaldehyde for 1 h, then permeabilized in 0.1% Triton X‐100 and 0.1% sodium citrate solution for 2 min on ice. Subsequently, 50 μL of TUNEL reaction mixture was added to each sample and cells were incubated for 1 h at 37°C. Cells were stained with DAPI. Images were obtained using a Zeiss AxioPlan II epifluorescence (FluoArc) microscope and processed with Axion Vision and Image J.^33^ DNA fragmentation was determined by evaluating the proportion of TUNEL‐positive stained cells.

### 2.14 PC‐normal fibroblast (NF) co‐culture model

To obtain PC‐conditioned media, PC cells were cultured to 50% confluence in DMEM supplemented with 10% FBS, then the media was changed to contain 1% FBS, 100 U/mL penicillin and 100 μg/mL streptomycin. After 48 h, the PC‐conditioned media was centrifuged and filtered before incubation with isolated NFs as described previously^21^ for 48 h. To establish the PC‐NF co‐culture model, PC cells and NFs were combined at a ratio of 2:1 and seeded into six‐well plates.

### 2.15 Immunohistochemical analysis

Sections (3‐µm thick) were deparaffinized, rehydrated and endogenous peroxidase activity was blocked via incubation with 3% hydrogen peroxide for 15 min at room temperature. Antigens were retrieved in a sodium citrate buffer (pH 6.0) for 3 min using a pressure cooker. Sections were blocked with 5% bovine serum albumin (BSA) for 30 min at room temperature to remove non‐specific binding. Then, sections were incubated with primary antibodies against HNRNPC (1: 200, Santa Cruz Biotechnology, sc‐32308, Dallas, TX, USA) and RhoA (1: 200, Abcam, ab54835, Cambridge, UK) overnight at 4°C. Next, sections were incubated with the secondary antibody for 1 h at 37°C. Finally, a chromogenic reaction was developed with DAB, and sections were counterstained with haematoxylin.

### 2.16 Immunofluorescence staining

Cells were washed with phosphate‐buffered saline (PBS), fixed with 4% formaldehyde and blocked with 1% BSA/PBS for 1 h at room temperature. Samples were then incubated with primary antibodies against α‐SMA (1: 250, Abcam, ab7817) and FAP (1: 200, Santa Cruz Biotechnology, sc‐65398) overnight at 4°C, followed by incubation with a Cy3‐conjugated secondary antibody (Beyotime, Nantong, China) for 1 h at room temperature. The cells were counterstained with DAPI to visualize the nuclei and examined using a confocal microscope (Olympus, Tokyo, Japan).

### 2.17 Xenograft studies in nude mice

Female BALB/c nude mice (16, aged 6–8 weeks) were obtained from Shanghai SLAC Laboratory Animal Co., Ltd. (Shanghai, China). The mice were maintained in pressurized ventilated cages according to institutional regulations. PanC‐1/NF co‐cultures were injected subcutaneously into the right flank of nude mice. The mice were divided into the following groups (four mice per group): control group: (1) PanC‐1‐siNC cells+NFs; (2) PanC‐1‐siHNRNPC cells+NFs; treatment group: (3) PanC‐1‐siNC cells+NFs+10 Gy; and (4) PanC‐1‐siHNRNPC cells+NFs+10 Gy. Mice were irradiated with a customized irradiator at a dose rate of 200 cGy/min and a linear accelerator (Varian, Palo Alto, CA) with a 10‐Gy X‐ray signal at a dose rate of 200 cGy/min on day 7.^34^ Animals were sacrificed and tumours were excised and frozen at −80°C or fixed in 10% formalin overnight. Routine histological examinations were then performed. The tumour volume was calculated as (length/2) × (width^2^).^21^ The animal experimental protocol was approved by the Animal Experiment Ethics Committee of Shanghai Jiao Tong University.

### 2.18 Statistical analysis

All values are presented as mean ± SEM. Statistical analysis was performed using SPSS 18.0 software (IBM, Chicago, IL, USA). Two‐tailed Student’s *t*‐test or one‐way ANOVA were used for data analysis. A *p*‐value of < 0.05 was considered statistically significant.

## 3 RESULTS

### 3.1 HNRNPC and RhoA are highly expressed in PC patient tissues

**FIGURE 1:**
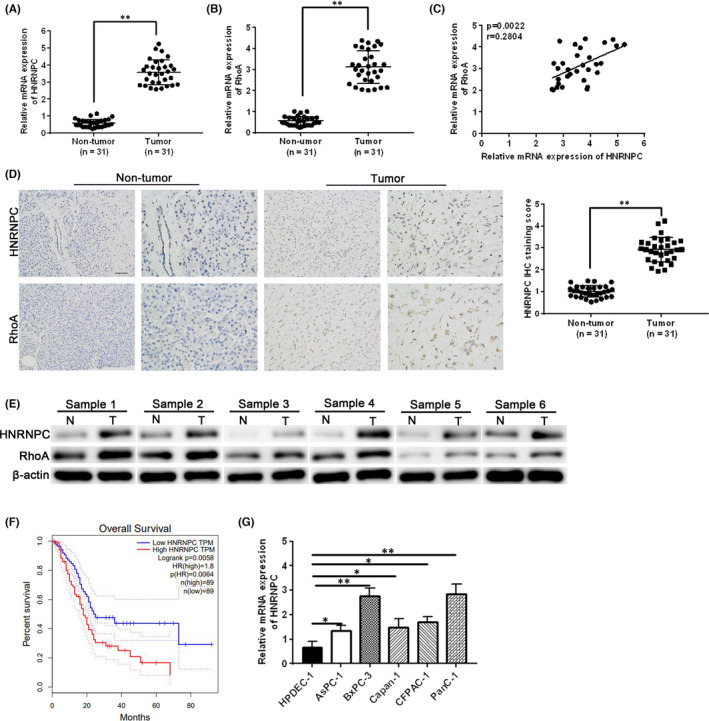
HNRNPC and RhoA expression levels are elevated in PC patient tumour tissue and PC cell lines. Relative HNRNPC (A) and RhoA (B) mRNA expression was measured in 31 pairs of PC patient tumour and corresponding adjacent non‐tumour tissues by RT‐PCR. (C) Correlation between HNRNPC and RhoA expression in PC tissues. (D) Immunohistochemical staining was used to examine HNRNPC and RhoA protein expression in PC patient tumour and corresponding adjacent non‐tumour tissue. Bar = 50 μm. Quantification of HNRNPC IHC staining score. (E) Western blot analysis of HNRNPC and RhoA protein expression in non‐tumours and tumours. (F) The correlation between HNRNPC expression and overall survival was determined by Kaplan‐Meier analysis (http://kmplot.com/analysis/index.php?p=service). (G) HNRNPC mRNA expression was measured in control human pancreatic ductal epithelial cells (HPDECs) and five PC cell lines. Data are presented as mean ± SEM. *p < 0.05 and ** *p* <0.01, using Student’s *t*‐test

To establish the relationship between HNRNPC, RhoA and radiation resistance in PC tissues, we examined HNRNPC and RhoA mRNA and protein expression levels by qRT‐PCR and Immunohistochemical (IHC) analysis in tumour and paired adjacent normal tissues from PC patients who received treatment at the Ruijin Hospital of Shanghai Jiao Tong University of China. Both HNRNPC and RhoA mRNA expression levels were significantly higher in PC patient tumour samples than the corresponding adjacent non‐tumour tissue (Figure 1A, B). Moreover, RhoA expression was positively correlated with HNRNPC expression (Figure 1C). IHC staining revealed that HNRNPC and RhoA protein expression levels were also significantly increased in the PC patient tumour samples compared to the adjacent normal tissue (Figure 1D, E). Kaplan‐Meier analysis (http://kmplot.com/analysis/index.php?p=service) indicated that the overall survival rate of PC patients was significantly lower in patients expressing high HNRNPC levels (Figure 1F). Finally, we demonstrated that HNRNPC mRNA expression was also significantly higher in multiple PC cell lines (AsPC‐1, BxPC‐3, Capan‐1, CFPAC‐1 and PanC‐1) compared to control human pancreatic ductal epithelial cells (HPDECs) (Figure 1G). Taken together, these data demonstrate that HNRNPC and RhoA are highly expressed in PC tissues and that high HNRNPC expression is associated with poor patient prognosis.

### 3.2 Abnormal HNRNPC expression is associated with the resistance of PC cells to radiation

**FIGURE 2:**
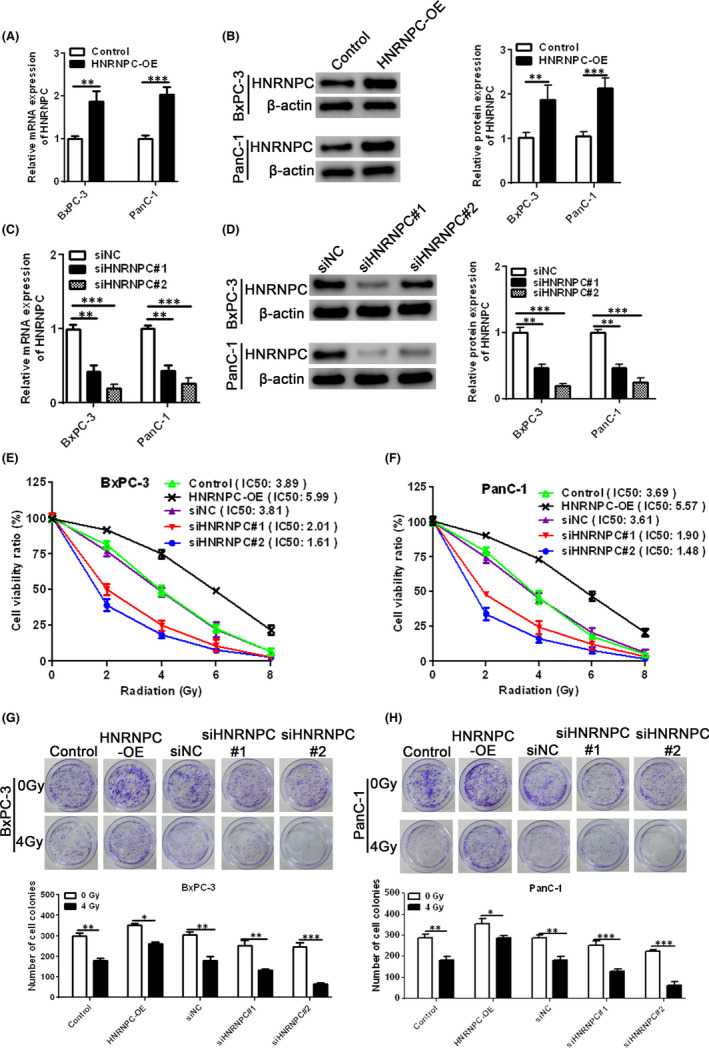
Effects of HNRNPC overexpression and silencing on radiation resistance in different PC cell lines. (A, B) BxPC‐3 and PanC‐1 cells were transfected with a HNRNPC‐overexpressing vector (HNRNPC‐OE) and the relative HNRNPC mRNA (A) and protein (B) expression levels were measured by qRT‐PCR and Western blotting, respectively. (C, D) Relative HNRNPC mRNA (C) and protein (D) expression levels were measured in BxPC‐3 and PanC‐1 cells transfected with siNC, siHNRNPC#1 or siHNRNPC#2 by qRT‐PCR and Western blotting, respectively. (E, F) The cell viability of HNRNPC‐OE or HNRNPC siRNA‐treated cells exposed to different doses of irradiation was measured by MTT assay. (G, H) Colony formation in BxPC‐3 and PanC‐1 cells treated with control, HNRNPC‐OE, siHNRNPC#1, siHNRNPC#2 or siNC and exposed to 4 Gy radiation. Data are presented as mean ± SEM, *n* = 3. *p < 0.05, ** *p* <0.01 and ***p < 0.001 using ANOVA

Next, we sought to determine whether HNRNPC gene expression affected the radiation sensitivity of different PC cell lines *in vitro* by overexpressing or silencing HNRNPC in PanC‐1 and BxPC‐3 cells. First, we used qRT‐PCR and Western blot analyses to determine the transfection efficiency of the HNRNPC overexpression vector (Figure 2A, B) and HNRNPC siRNA (Figure 2C, D) in different cell lines. Our MTT data demonstrated that HNRNPC overexpression improved cell viability, while HNRNPC interference led to a decrease in cell viability in BxPC‐3 and PanC‐1 cell lines after exposure to different doses of irradiation (Figure 2E, F). Consistent with these findings, colony formation was significantly higher in BxPC‐3 and PanC‐1 cells overexpressing HNRNPC than HNRNPC‐silenced cells after exposure to 4 Gy radiation (Figure 2G, H). Thus, taken together, our findings suggest that HNRNPC overexpression promotes radiation resistance in PC cells, while HNRNPC knockdown increases radiosensitivity.

### 3.3 Radiation resistance induced by HNRNPC overexpression is mediated by RhoA

**FIGURE 3:**
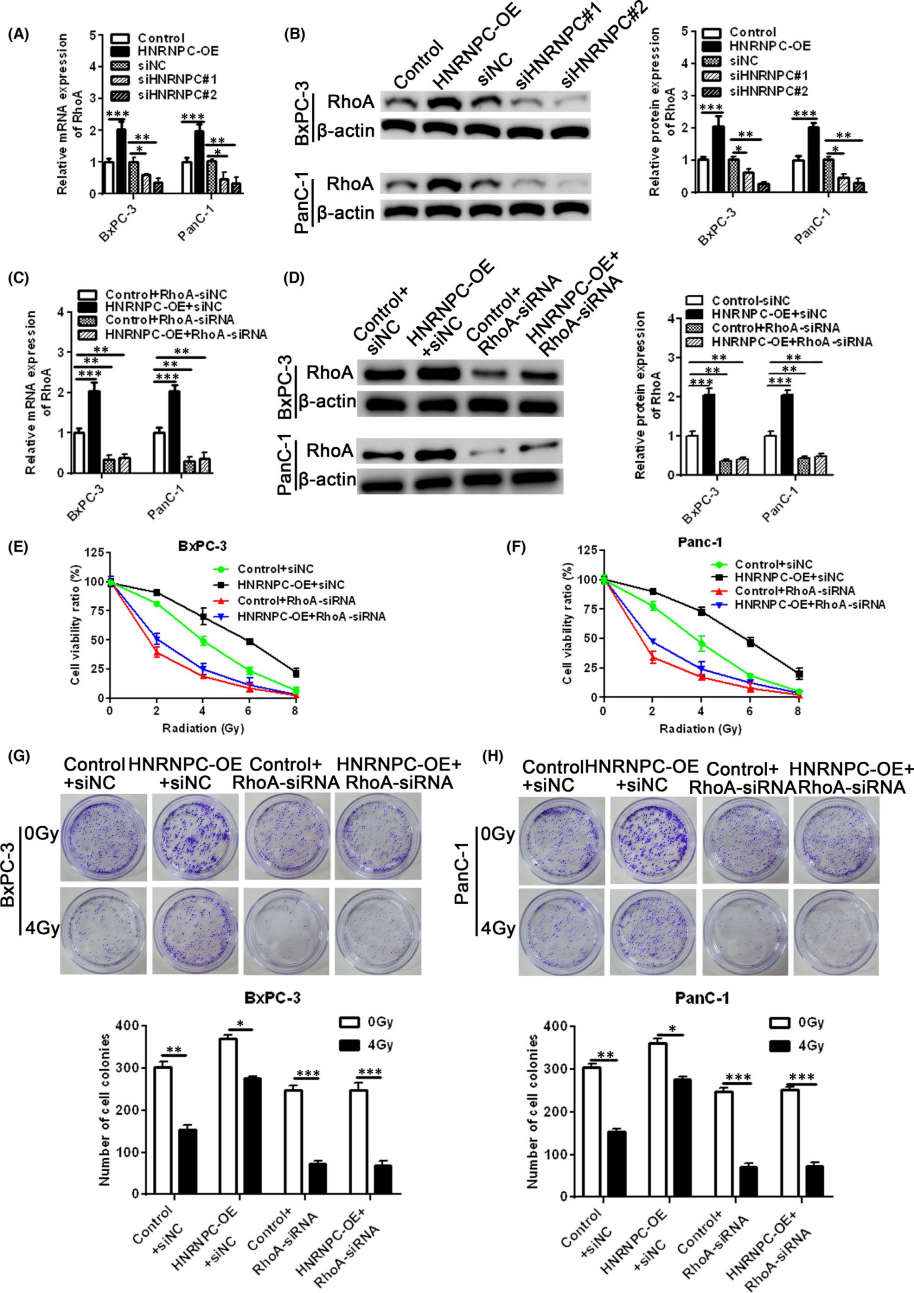
HNRNPC affects cell proliferation and radiation resistance via RhoA. (A, B) BxPC‐3 and PanC‐1 cells were transfected with a HNRNPC‐overexpressing vector (HNRNPC‐OE) or HNRNPC siRNA and the relative Rho mRNA (A) and protein (B) expression levels were measured by qRT‐PCR and Western blotting, respectively. (C, D) BxPC‐3 and PanC‐1 cells were treated with siNC, siNC+HNRNPC‐OE, siRhoA or HNRNPC‐OE+siRhoA and the relative RhoA mRNA (C) and protein (D) expression levels were measured by qRT‐PCR and Western blotting, respectively. (E, F) MTT assay was used to measure cell viability in BxPC‐3 (E) and PanC‐1 (F) cells treated with siNC, siNC+HNRNPC‐OE, siRhoA or HNRNPC‐OE+siRhoA and exposed to different irradiation intensities. (G‐H) Colony formation assay was performed in BxPC‐3 (G) and PanC‐1 (H) cells treated with siNC, siNC+HNRNPC‐OE, siRhoA or HNRNPC‐OE+siRhoA and exposed to 4 Gy irradiation. Data are given as mean ± SEM, *n* = 3. *p < 0.05, **p < 0.01, ***p < 0.001 and ****p < 0.0001 using ANOVA

Using Starbase (http://starbase.sysu.edu.cn/index.php), we predicted that HNRNPC interacted with RhoA. To further examine the relationship between HNRNPC and RhoA, we measured RhoA mRNA and protein expression in HNRNPC‐overexpressing and ‐silenced cells using qRT‐PCR and Western blot analysis. We found that RhoA mRNA and protein expression levels were significantly increased in cells overexpressing HNRNPC, while knockdown of HNRNPC led to a significant reduction in RhoA (Figure 3A, B). Thus, RhoA expression was positively correlated with HNRNPC expression. Next, we confirmed the transfection efficiency of RhoA siRNA in siNC, HNRNPC‐OE+siNC, siRhoA and HNRNPC‐OE+siRhoA (Figure 3C, D). We found that RhoA interference reduced the cell viability of control and HNRNPC‐overexpressing BxPC‐3 and PanC‐1 cell lines after exposure to various doses of irradiation (Figure 3E, F). Similarly, RhoA interference reduced the colony‐forming ability of control and HNRNPC‐overexpressing BxPC‐3 and PanC‐1 cell lines after exposure to 4 Gy irradiation (Figure 3G, H **)**. Taken together, our findings suggest that RhoA mediates radiation resistance induced by HNRNPC overexpression.

### 3.4 RhoA is a downstream target of HNRNPC

**FIGURE 4:**
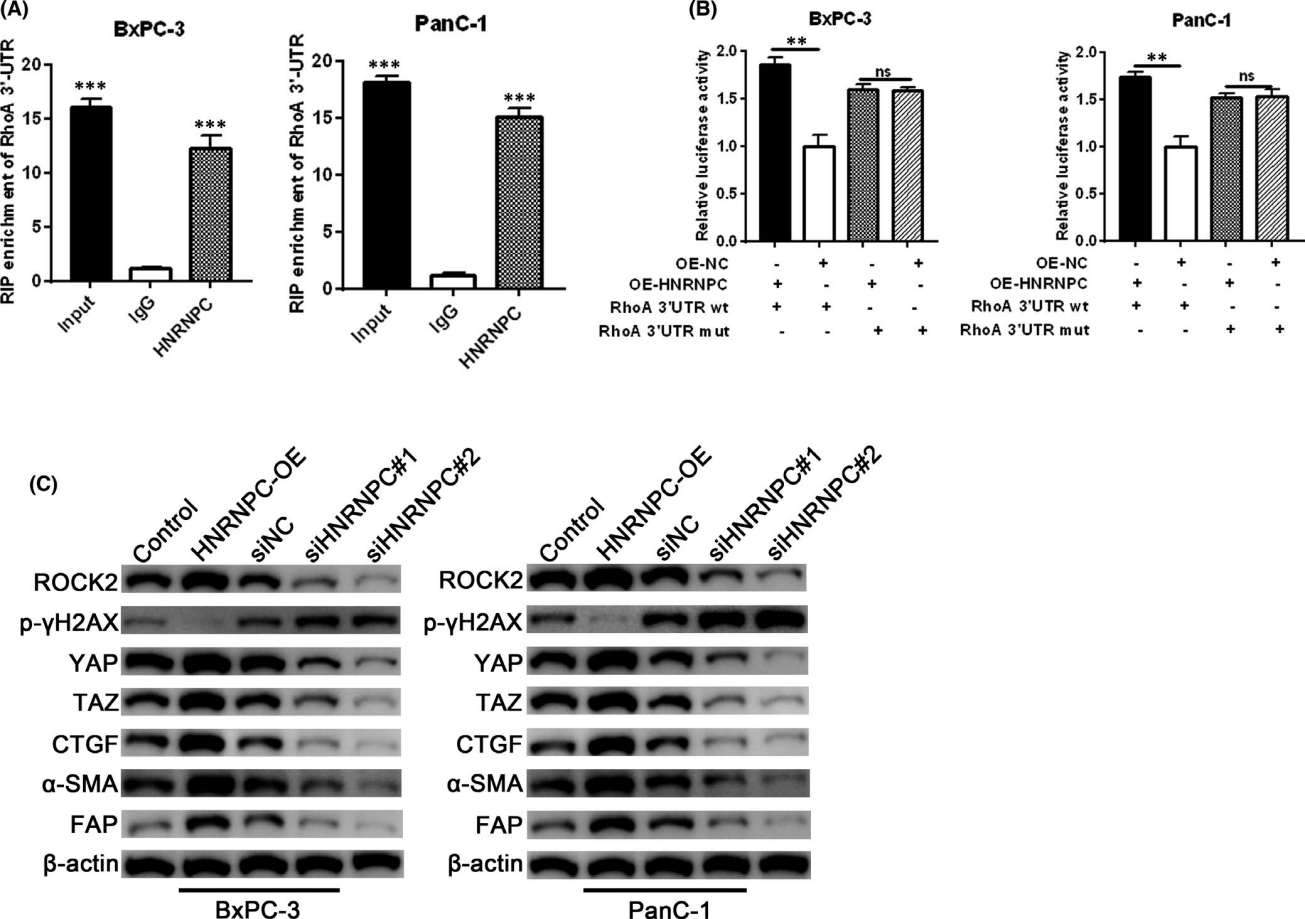
RhoA is a downstream target of HNRNPC. (A) The interaction between HNRNPC and RhoA was examined by RNA immunoprecipitation. (B) Luciferase reporter assay was used to detect HNRNPC binding to RhoA 3’UTR. (C) The protein levels of downstream target genes of RhoA after HNRNPC knockdown or overexpression in BxPC‐3 and PanC‐1 cells were determined by Western blotting. Data are given as mean ± SEM, *n* = 3. *p < 0.05, **p < 0.01 and ***p < 0.001, using ANOVA

As HNRNPC is an RNA binding protein, we next sought to determine whether RhoA was a direct target of HNRNPC binding. We confirmed that HNRNPC is bound to RhoA mRNA by RIP assay (Figure 4A). Consistent with these findings, significantly increased luciferase activity was observed in HNRNPC‐overexpressing BxPC‐3 and PanC‐1 cells co‐transfected with wild‐type RhoA 3′UTR WT, but not mutated RhoA 3′UTR mut (Figure 4B). Next, we investigated the signalling pathways downstream of HNRNPC‐induced RhoA. Since RhoA has recently been implicated in fibrosis through the activation of the ROCK/YAP/TAZ axis,^23^ we examined the effects of HNRNPC overexpression and silencing on the mRNA and protein expression of these pathways. We found that HNRNPC overexpression led to increased ROCK2, YAP and TAZ protein (Figure 4C) and mRNA (Supporting Information Figure S1) expression, while HNRNPC knockdown had the opposite effect, suggesting that HNRNPC mediates its effect via the RhoA/ROCK2‐YAP/TAZ axis. Consistent with these changes in YAP/TAZ expression, we found increased expression of the YAP/TAZ downstream targets, CTGF, α‐SMA and FAP in HNRNPC‐OE‐treated cells,^29^, ^35^ while HNRNPC siRNA treatment led to reduced expression (Figure 4C, Supporting Information Figure S1). Taken together, our findings demonstrate that RhoA is a downstream target of HNRNPC, which acts through the RhoA/ROCK2‐YAP/TAZ axis to promote fibrosis.

### 3.5 Inhibition of the RhoA/ROCK2 signalling pathway reduces DNA damage repair and radiation resistance

**FIGURE 5:**
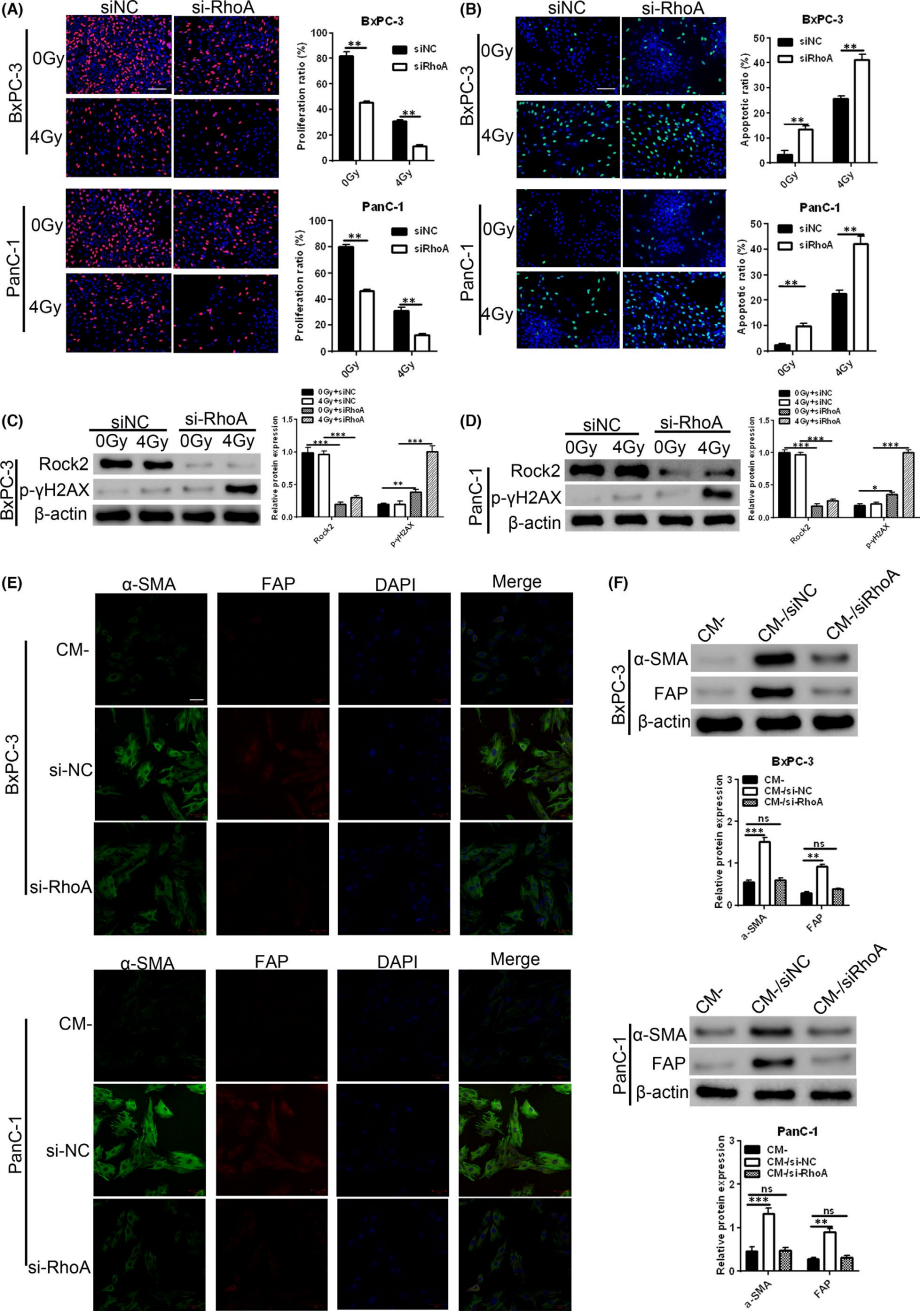
Inhibition of the RhoA/ROCK2 pathway reduces DNA damage repair and radiation resistance. (A) EdU staining was used to measure DNA synthesis in BxPC‐3 and PanC‐1 cells treated with siRhoA or siNC and exposed to 4 Gy irradiation, bar = 100 μm. (B) TUNEL staining was used to evaluate apoptosis in BxPC‐3 and PanC‐1 cells treated with siRhoA or siNC and exposed to 4 Gy irradiation, bar = 100 μm. (C, D) Western blot analysis of ROCK2 and p‐γH2AX expression in BxPC‐3 and PanC‐1 cells treated with siRhoA or siNC and exposed to 4 Gy irradiation. (E, F) NFs were cultured with conditioned media obtained from si‐NC and siRhoA‐treated BxPC‐3 and PanC‐1 cells. Immunofluorescence staining of activated fibroblast markers, FAP and α‐SMA, bar = 50 μm (E). Western blot analysis of FAP and α‐SMA protein expression (F). Data are given as mean ± SEM, *n* = 3. *p < 0.05, **p < 0.01, ***p < 0.001 and ****p < 0.0001 using ANOVA

Radiation resistance is associated with increased DNA repair.^36^ To determine whether HNRNPC regulates DNA repair mechanisms, we examined expression of phosphorylated γH2AX, a marker of DNA double‐strand breaks. We found increased levels of p‐γH2AX after HNRNPC knockdown, while HNRNPC‐OE treatment led to decreased p‐γH2AX expression (Figure 4C, Supplementary Figure 1). To further elucidate the role of HNRNPC and RhoA in mediating radiation resistance in PC cells, we examined the effects of silencing RhoA expression on proliferation and DNA damage repair in PC cells exposed to irradiation. EdU staining revealed that RhoA interference reduced DNA synthesis and cell proliferation in BxPC‐3 and PanC‐1 cells in both control (0 Gy) and irradiated (4 Gy) cells (Figure 5A). TUNEL staining demonstrated that RhoA knockdown increased DNA fragmentation and apoptosis in both control (0 Gy) and irradiated (4 Gy) cells (Figure 5B). RhoA silencing also led to a significant decrease in ROCK2 protein expression levels, while p‐γH2AX levels were significantly increased especially after irradiation (Figure 5C, D). Taken together, our findings show that inhibition of the RhoA/ROCK2 pathway leads to decreased proliferation, increased apoptosis and increased DNA damage after radiation treatment, suggesting that RhoA has a role in mediating radiation resistance in PC cells via the RhoA/ROCK2‐YAP/TAZ signalling pathways.

### 3.6 RhoA inhibition can hinder CAF activity and weaken radiation resistance in PC

Since we observed increased expression of fibrotic markers in PC cells over‐expressing HNRNPC, we next examined the effects of tumour cell supernatants on CAFs. We established a cell co‐culture system using PC cell‐conditioned media and NFs to examine the role of RhoA in CAF activation. Immunofluorescence staining revealed that culturing NFs in conditioned media obtained from RhoA‐siRNA‐treated BxPC‐3 and PanC‐1 cells led to a decrease in the expression of the CAF‐related proteins α‐SMA and FAP compared to control si‐NC‐treated cells (Figure 5E). Similar results were observed by Western blot analysis (Figure 5F). Together, these findings suggest that RhoA has a role in regulating CAF activation.

**FIGURE 6:**
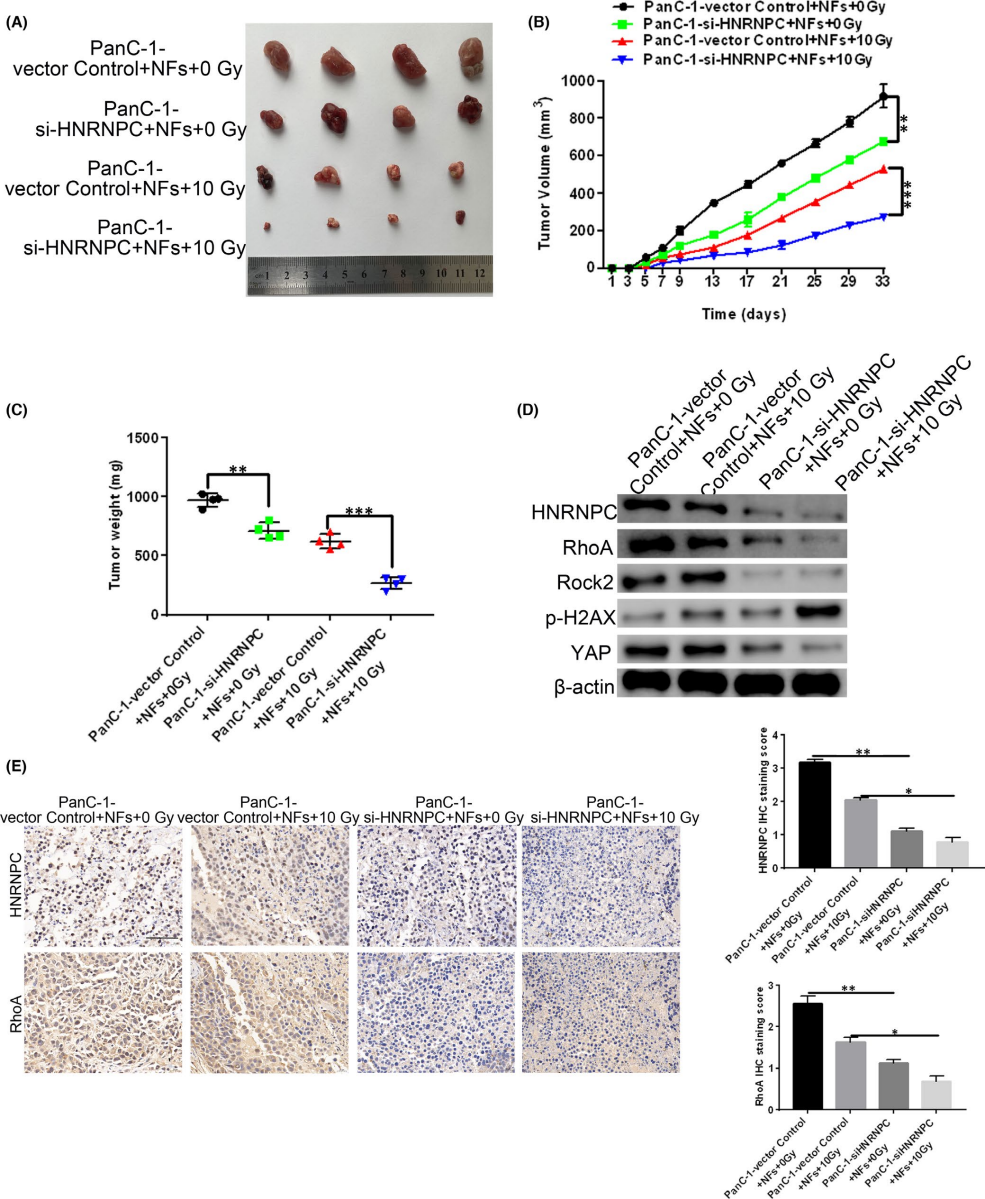
PanC‐1/NF co‐cultures enhance radiation resistance, while HNRNPC knockdown in mice reduces radiation resistance. A xenograft tumour model was established in nude mice using PanC‐1/NF co‐cultured cells, in which the PanC‐1 cells had been treated with either si‐NC or si‐HNRNPC. Tumours were exposed to 0 Gy or 10 Gy radiation. (A) Representative tumour images. (B) Tumour volume. (C) Tumour weight. (D) Western blot analysis of HNRNPC, RhoA, ROCK2, H2AX and YAP protein expression in tumours. (E) Immunohistochemical staining of HNRNPC and RhoA expression in tumours and the quantitative results of immunohistochemical staining. Bar = 20 μm. Data are given as mean ± SEM, *n* = 4. **p < 0.01 and ***p < 0.001

Next, we established a xenograft tumour model in nude mice to determine the effects of HNRNPC silencing on the radiation resistance of PC cells in vivo. NFs and control or si‐HNRNPC‐treated PanC‐1‐cells were co‐cultured, then subcutaneously injected into nude mice. We found that mice injected with siNC‐treated PanC‐1/NF co‐cultures developed large tumours (Figure 6A‐C), which were significantly decreased after exposure to 10 Gy irradiation. Tumours that developed from NFs co‐cultured with HNRNPC‐silenced PanC‐1 cells were significantly smaller, and irradiation of these PanC1/NF co‐cultures with 10 Gy led to the most significant decrease in tumour growth (Figure 6A‐C). These findings suggest that silencing HNRNPC increases the radiation sensitivity of PC cells. Western blot analysis of the tumour samples from mice injected with siNC‐treated PanC‐1/NF co‐cultures revealed that exposure to 10 Gy irradiation resulted in a decrease in HNRNPC and RhoA protein expression, as well as increased p‐γH2AX levels (Figure 6D). Tumour cells from mice injected with siHNRNPC‐silenced PanC‐1/NF co‐cultures had significantly decreased HNRNPC, RhoA, ROCK2 and YAP expression, suggesting that radiation resistance is mediated via HNRNPC and the downstream RhoA/ROCK2/YAP signalling pathway in PC. Consistent with these findings, DNA damage following irradiation, as measured by p‐γH2AX expression, was significantly higher in tumours from mice injected with siHNRNPC‐treated PanC‐1/NF co‐cultures. Similarly, immunohistochemical staining with antibodies against HNRNPC and RhoA confirmed the Western blot results (Figure 6E). Thus, our findings show that knockdown of HNRNPC in PanC‐1 cells increases radiation sensitivity through the RhoA/ROCK2/YAP signalling pathway.

## 4 DISCUSSION

PC is a lethal cancer that is asymptomatic in its early stages and has non‐specific symptoms in later stages, making it difficult to diagnose.^1^ GLOBOCAN 2018 statistics indicated a total of 458 918 new PC cases reported in 2018 and estimated that 355 317 new cases will occur before 2040.^1^ Therefore, understanding the underlying mechanisms of PC tumorigenesis and progression is critical for the development of effective treatment strategies. HNRNPC belongs to a family of HNRNPs, which are RNA‐binding proteins that participate in many aspects of nucleic acid metabolism and gene expression regulation and thus tumour development.^37^ Previous studies have described elevated levels of HNRNPC in some cancer cells, while HNRNPC knockdown has been shown to slow down cell proliferation and tumour growth in breast cancer.^12^ HNRNPC has also been identified as a key regulator of metastatic potential in glioblastoma cells.^38^

Since PC cells generally develop radiation resistance during treatment,^39^ determining the mechanisms underlying radioresistance is critical for the development of future treatment strategies. The present study examined radiation‐resistant PC tissue samples from clinical patients for both HNRNPC and RhoA levels, which are involved in the proliferation and migration of tumour cells.^40^ Previously, an association between the mRNA‐binding protein hnRNP‐Q1 and RhoA has been reported.^14^ Here, we found that both HNRNPC and RhoA are expressed in radiation‐resistant PC tissues. In addition, we demonstrated that RhoA was a downstream target of HNRNPC. Previous studies have suggested that HNRNPC is associated with the coordination of DNA‐damage responses and radiation‐induced apoptosis pathways.^41^ Here, we found that overexpression of HNRNPC was associated with increased radioresistance, while knockdown of HNRNPC led to increased PC sensitivity. Furthermore, inhibition of RhoA attenuated radiation resistance caused by HNRNPC overexpression.

The RhoA/ROCK pathway has been implicated in dynamic crosstalk regulation between cancer cells and their microenvironment, which may be used to inhibit cancer metastatic processes.^42^, ^43^ Rho inhibition has been shown to decrease tumour cell survival after radiation treatment, while ROCK inhibitors, in particular, may enhance chemo‐ and radiotherapy efficacy.^44^ Rho/ROCK inhibitors may also act as pro‐vascular agents to improve tumour blood flow and increase cell exposure to chemotherapy or even sensitize cells to the radiation effects.^45^ Although a potential role for RhoA, which has been shown to regulate glioblastoma radioresistance via survivin,^46^ and ROCK2 in radiation resistance has been suggested, their collective mechanism remains poorly understood.^18^ PC radiosensitivity has also been enhanced via the YAP/TAZ signalling pathway.^47^ Growing evidence suggests that YAP promotes resistance to various anti‐cancer therapies, including radiation therapy.^48^ Our results show that HNRNPC mediates radiation resistance through the RhoA/ROCK‐YAP/TAZ pathways and identifies HNRNPC as a potential target to sensitize PC to radiation.

The regulatory effects of RhoA/ROCK2 and RhoA/YAP on PC radiation resistance were also investigated in the present study. Inhibition of the RhoA/ROCK2 pathway reduced DNA damage repair and radiation resistance. Previous studies have demonstrated that CAFs participate in cytoskeletal alteration via the RhoA/ROCK pathway and increased YAP localization.^28^ YAP fibroblast activity remodelled matrix organization and increased matrix stiffness, promoting cancer cell migration and invasion.^18^ Here, we found that HNRNPC or RhoA inhibition limited the activity of CAFs and weakened PC radiation resistance. Overall, our study identified the HNRNPC‐RhoA/ROCK2‐YAP/TAZ axis in PC radiation resistance, providing a potential therapeutic target for improving PC treatment.

There are some limitations associated with this study. First, only two regulatory pathways of HNRNPC in PC radiation resistance were explored. Other regulatory signaling pathways may be involved and should be investigated in the future. Second, this work did not conduct an in‐depth research on the two identified signalling pathways. Thus, more detailed studies are required to make definitive conclusions about the role of these pathways in radiation resistance. Tumour‐associated fibroblasts are an important part of cancer development and treatment, while tumour radiation resistance is a major problem in tumour radiotherapy. In order to clarify the relationship between CAFs and tumour radiation resistance in the future, the regulatory mechanism for CAFs in PC cell radiation resistance should be investigated in greater detail to provide more therapeutic targets and explore additional directions for cancer treatment.

In summary, we have shown that inhibition of the HNRNPC‐RhoA/ROCK2‐YAP/TAZ axis can potentially sensitize PC cells to radiation, providing a novel method to enhance the treatment of PC.

## CONFLICT OF INTEREST

The authors confirm that there are no conflicts of interest.

## AUTHOR CONTRIBUTIONS

NX, WH and ZMW designed the research study. NX, NNY and QGS conceived and carried out experiments. YJC and JL collected fresh samples and performed the follow‐up for the patients. XYL coordinated the tissue collection. NX, NNY, QGS ZYW, XYL, YJC and JL analysed data. NX, NNY, QGS, WH and ZMW wrote and/or reviewed the manuscript. All authors were involved in writing the paper and had final approval of the submitted and published versions.

## AUTHORS CONTRIBUTION

**Ning Xia:** Conceptualization (equal); Data curation (equal); Formal analysis (equal); Investigation (equal); Methodology (equal); Project administration (equal); Writing – original draft (equal). **Nannan Yang:** Data curation (equal); Methodology (equal); Resources (equal); Validation (equal); Writing – original draft (equal). **Qungang Shan:** Data curation (lead); Methodology (equal); Resources (equal); Validation (equal); Writing – original draft (lead). **Ziyin Wang:** Data curation (supporting); Formal analysis (equal); Investigation (equal); Methodology (supporting). **Xiaoyu Liu:** Data curation (supporting); Methodology (supporting); Resources (supporting). **Yingjie Chen :** Conceptualization (supporting); Data curation (supporting); Resources (supporting). **Jian Lu:** Methodology (supporting); Resources (supporting); Validation (supporting). **Wei Huang:** Funding acquisition (equal); Writing – review & editing (equal). **Zhongmin Wang:** Funding acquisition (equal); Writing—review & editing (equal).

## Supporting information

FIGURE S1 The mRNA levels of downstream target genes of RhoA after HNRNPC knockdown or overexpression in BxPC‐3 and PanC‐1 were examined by qRT‐PCR

## Data Availability

The data that support the findings of this study are available from the corresponding author upon reasonable request.
