# Supplementary figures and images for: HNRNPC regulates RhoA to induce DNA damage repair and cancer‐associated fibroblast activation causing radiation resistance in pancreatic cancer
Source: J Cell Mol Med. 2022 Mar 11;26(8):2322–36. doi: 10.1111/jcmm.17254 (PMC8995438; doi:10.1111/jcmm.17254)

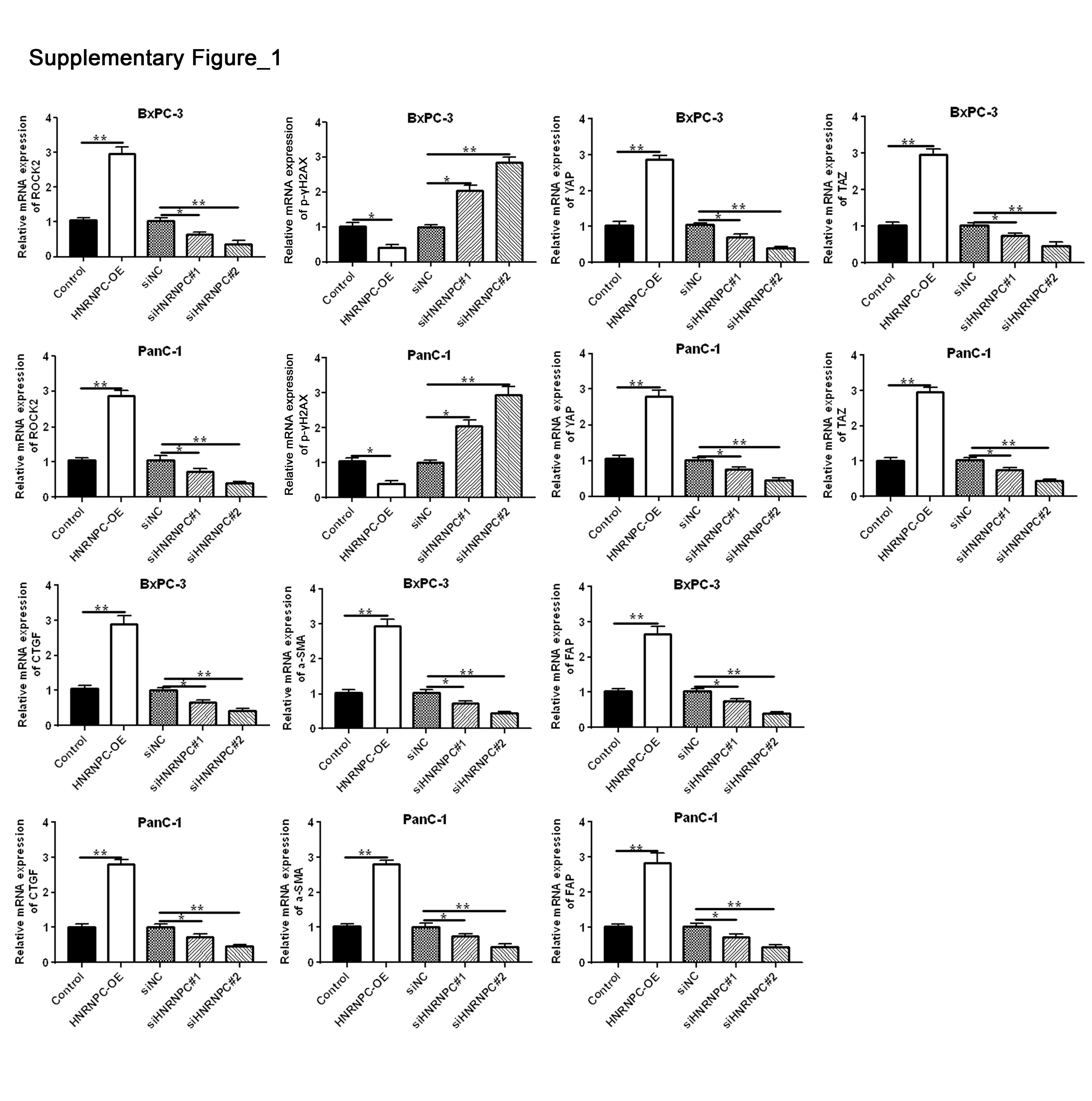

Supplement: Supplementary file 1 — FIGURE S1 The mRNA levels of downstream target genes of RhoA after HNRNPC knockdown or overexpression in BxPC‐3 and PanC‐1 were examined by qRT‐PCR [file JCMM-26-2322-s001.tif]
